# Floquet Second-Order Topological Phases in Momentum Space

**DOI:** 10.3390/nano11051170

**Published:** 2021-04-29

**Authors:** Longwen Zhou

**Affiliations:** Department of Physics, College of Information Science and Engineering, Ocean University of China, Qingdao 266100, China; zhoulw13@u.nus.edu

**Keywords:** topological insulators, topological phase transition, floquet system

## Abstract

Higher-order topological phases (HOTPs) are characterized by symmetry-protected bound states at the corners or hinges of the system. In this work, we reveal a momentum-space counterpart of HOTPs in time-periodic driven systems, which are demonstrated in a two-dimensional extension of the quantum double-kicked rotor. The found Floquet HOTPs are protected by chiral symmetry and characterized by a pair of topological invariants, which could take arbitrarily large integer values with the increase of kicking strengths. These topological numbers are shown to be measurable from the chiral dynamics of wave packets. Under open boundary conditions, multiple quartets Floquet corner modes with zero and π quasienergies emerge in the system and coexist with delocalized bulk states at the same quasienergies, forming second-order Floquet topological bound states in the continuum. The number of these corner modes is further counted by the bulk topological invariants according to the relation of bulk-corner correspondence. Our findings thus extend the study of HOTPs to momentum-space lattices and further uncover the richness of HOTPs and corner-localized bound states in continuum in Floquet systems.

## 1. Introduction

Higher-order topological phases (HOTPs) in *D* spatial dimensions are characterized by symmetry-protected states localized along its (D−n)-dimensional boundaries, where 1<n≤D [1,2,3,4,5,6,7,8]. The presence of these unique topological matter is usually guaranteed by the coexistence of crystal and non-spatial symmetries, and their classifications go beyond the tenfold way of first-order topological insulators and superconductors [9,10,11,12]. Besides great theoretical efforts in the study of higher-order topological insulators [13,14,15,16,17,18,19,20,21,22,23,24,25,26,27,28,29,30,31,32], superconductors [33,34,35,36,37,38,39,40,41,42,43,44,45,46,47,48] and semimetals [49,50,51,52,53,54], HOTPs have also been observed in solid state materials [55,56,57,58,59], photonic waveguides [60,61,62,63,64,65,66,67], acoustic systems [68,69,70,71,72,73,74,75], electrical circuits [76,77,78,79,80] and superconducting qubits [81], leading to potential applications such as acoustic sensing [68,69] and holonomic quantum computation [48].

In recent years, the study of HOTPs has been generalized to nonequilibrium systems, such as those subject to time-periodic drivings [82,83,84,85,86,87,88,89,90,91,92,93,94] or non-Hermitian effects [95,96,97,98,99,100,101,102,103]. The motivation behind the exploration of HOTPs in periodically driven systems is threefold. First, driving fields could induce symmetries and phase transitions that are unique to Floquet systems [104,105], yielding Floquet HOTPs with topological properties that go beyond any static counterparts [82,93]. Second, periodic driving fields could in general enlarge the range of hoppings in a lattice [106], creating Floquet HOTPs with large topological numbers and many topological corner/hinge modes [82], which have potential applications in the construction of topological time crystals and Floquet quantum computing schemes [83]. Third, under appropriate conditions, certain Floquet systems could form lattice structures in momentum space, whose topological properties are of intrinsic dynamical origins. Intriguing phenomena related to such momentum space topology including the quantized acceleration as an analog of the topological Thouless pump [107] and the integer quantum Hall effects from chaos [108]. The first two aspects have led to the discoveries of various Floquet HOTPs in both Hermitian and non-Hermitian systems [82,83,84,85,86,87,88,89,90,91,92,93,94,95]. However, the momentum-space counterpart of Floquet HOTPs and their topological characterizations have rarely been explored.

In this manuscript, we investigate a periodically kicked rotor in two spatial dimensions, whose momentum space could form a two-dimensional (2D) discrete lattice holding rich Floquet HOTPs. In Section 2, we introduce the Hamiltonian of the system and obtain its Floquet operator under the quantum resonance condition. Based on the symmetry analysis of the model, we construct a pair of integer topological invariants $\left(w_{0},w_{\pi }\right)$ in Section 3, which could fully characterize the Floquet HOTPs that are protected by the chiral symmetry of the system. These Floquet HOTPs are further shown to be able to possess arbitrary large topological numbers with the increase of kicking strengths. In Section 4, we show that these topological invariants could be dynamically probed by measuring the time-averaged mean chiral displacements of wave packets in two-dimension. Under the open boundary conditions, we find many quartets of Floquet corner modes at zero and π quasienergies in Section 5. The numbers of these corner modes are predicted by the bulk topological invariants $\left(w_{0},w_{\pi }\right)$, yielding the bulk-corner correspondence of Floquet HOTPs in momentum space. Moreover, the zero and π Floquet corner modes are found to be embedded in the continuous bulk bands of delocalized states, forming corner-localized Floquet bound states in the continuum that are originated from higher-order Floquet topology. We summarize our results and discuss potential future directions in Section 6.

## 2. Model

In this section, we introduce a representative driven lattice model, which could possess rich Floquet HOTPs in momentum space. Our system can be viewed as a two-dimensional extension of the double kicked rotor (or lattice) model [109,110,111,112], which describes a quantum particle kicked twice by a periodic potential at different times within each driving period. The time-dependent Hamiltonian of the system takes the form

(1) $$\hat{H}={\hat{H}}_{0}+\hat{V}\sum_{\ell \in Z}\delta \left(\frac{t}{T}-\ell \right)+\hat{W}\sum_{\ell \in Z}\delta \left(\frac{t-\tau }{T}-\ell \right),$$

where

(2) $${\hat{H}}_{0}=\frac{{\hat{p}}_{x}^{2}+{\hat{p}}_{y}^{2}}{2},$$

(3) $$\hat{V}=\kappa_{1}\cos\left(\hat{x}+\phi_{x}\right)+\kappa_{3}\cos\left(\hat{y}+\phi_{y}\right),$$

and

(4) $$\hat{W}=\kappa_{2}\cos\left(\hat{x}\right)+\kappa_{4}\cos\left(\hat{y}\right).$$

Here, $\left(\hat{x},\hat{y}\right)$ and $\left({\hat{p}}_{x},{\hat{p}}_{y}\right)$ are the position and momentum operators of the particle along *x* and *y* directions. *T* is the driving period. τ∈(0,T) controls the time delay between the two kicks inside a driving period. $\kappa_{1,3}$ and $\kappa_{2,4}$ are kicking strengths of the potentials along *x* and *y* directions. $\phi_{x,y}\in \left[0,2\pi \right)$ describe the phase differences between the kicking potentials applied at t=ℓT and t=ℓT+τ in the *ℓ*’s driving period. The quantities in Equations (2)–(4) are all set in dimensionless units. In experiments, the model Hamiltonian $\hat{H}$ may be realized in cold atom systems, where the kicking potentials could be implemented by optical-lattice potentials with relative phase shifts [111,112]. Within a given driving period (e.g., from $t=0^{-}$ to $t=T+0^{-}$), the dynamics of the system is therefore governed by a kick $\hat{V}$ applied at t=0, followed by the free evolution ${\hat{H}}_{0}$ from t=0→τ, a second kick $\hat{W}$ at t=τ, and finally the free evolution ${\hat{H}}_{0}$ over a time duration T−τ. The Floquet operator, which describes the evolution of the system over such a complete driving period, is then given by

(5) $$\hat{U}=e^{-i\frac{T-\tau }{\hbar }{\hat{H}}_{0}}e^{-i\frac{T}{\hbar }\hat{W}}e^{-i\frac{\tau }{\hbar }{\hat{H}}_{0}}e^{-i\frac{T}{\hbar }\hat{V}}.$$

Due to the periodicity of kicking potentials $\hat{V}$ and $\hat{W}$ in $\hat{x}$ and $\hat{y}$, the eigenvalues of momentum operators ${\hat{p}}_{x,y}$ take the forms $\hbar (n_{x,y}+k_{x,y})$, where $n_{x,y}\in Z$, and $k_{x,y}\in \left[0,1\right)$ are the quasimomenta. A Floquet system with periodicity in momentum space is achieved by setting $k_{x,y}=0$, which maybe realized experimentally by a Bose–Einstein condensate with large coherence width [113,114,115,116]. Under this condition, we can identify the momentum operators ${\hat{p}}_{x,y}$ in Equation (2) as $\hbar {\hat{n}}_{x,y}$ with integer eigenvalues $n_{x,y}$. The Floquet operator in Equation (5) then takes the explicit form

(6) $$\hat{U}=e^{-i\hbar \left(T-\tau \right)\frac{{\hat{n}}_{x}^{2}+{\hat{n}}_{y}^{2}}{2}}e^{-i[K_{2}\cos\left(\hat{x}\right)+K_{4}\cos\left(\hat{y}\right)]}e^{-i\hbar \tau \frac{{\hat{n}}_{x}^{2}+{\hat{n}}_{y}^{2}}{2}}e^{-i[K_{1}\cos\left(\hat{x}+\phi_{x}\right)+K_{3}\cos\left(\hat{y}+\phi_{y}\right)]},$$

where we introduce $K_{j}=\kappa_{j}T/\hbar$ for j=1,2,3,4 as rescaled dimensionless kicking strengths. Furthermore, under the quantum resonance condition ℏT=4π that has been considered experimentally [113,114,115,116,117,118,119], we obtain the two-dimensional extension of on-resonance double kicked rotor (or lattice) model, whose Floquet operator reads

(7) $$\hat{U}=e^{i\hbar \tau \frac{{\hat{n}}_{x}^{2}+{\hat{n}}_{y}^{2}}{2}}e^{-i[K_{2}\cos\left(\hat{x}\right)+K_{4}\cos\left(\hat{y}\right)]}e^{-i\hbar \tau \frac{{\hat{n}}_{x}^{2}+{\hat{n}}_{y}^{2}}{2}}e^{-i[K_{1}\cos\left(\hat{x}+\phi_{x}\right)+K_{3}\cos\left(\hat{y}+\phi_{y}\right)]}.$$

It is then clear that, once ℏτ=2πp/q, with *p* and *q* being coprime integers, the Floquet operator $\hat{U}$ will have translational symmetries in both ${\hat{n}}_{x}$ and ${\hat{n}}_{y}$ with the common period *q*, i.e., a periodic crystal structure in the momentum space of the two-dimensional on-resonance double-kicked lattice. In one-dimensional (1D) descendant models of Equation (7), rich first-order Floquet topological phases have been discovered, which are characterized by large Chern (winding numbers), multiple chiral (dispersionless) edge modes and topologically quantized acceleration in momentum space [107,120,121,122,123]. These discoveries further motivate us to explore HOTPs in the 2D on-resonance double-kicked lattice model.

To obtain a minimal version of our model with nontrivial higher-order topology, we choose the time delay between the two kicks to be τ=T/4, which implies that ℏτ=π. Moreover, fixing the phase differences at $\phi_{x}=\phi_{y}=\pi /2$, the Floquet operator of the 2D on-resonance double-kicked lattice reduces to

(8) $$\hat{U}=e^{i\frac{\pi }{2}\left({\hat{n}}_{x}^{2}+{\hat{n}}_{y}^{2}\right)}e^{-i[K_{2}\cos\left(\hat{x}\right)+K_{4}\cos\left(\hat{y}\right)]}e^{-i\frac{\pi }{2}\left({\hat{n}}_{x}^{2}+{\hat{n}}_{y}^{2}\right)}e^{i[K_{1}\sin\left(\hat{x}\right)+K_{3}\sin\left(\hat{y}\right)]}.$$

Since $\left[{\hat{n}}_{x},\hat{y}\right]=\left[{\hat{n}}_{y},\hat{x}\right]=0$, the 2D on-resonance double-kicked lattice can be viewed as two 1D kicked lattice models lying along two different spatial dimensions, i.e., $\hat{U}={\hat{U}}_{x}⊗{\hat{U}}_{y}$, where

(9) $${\hat{U}}_{x}=e^{i\frac{\pi }{2}{\hat{n}}_{x}^{2}}e^{-iK_{2}\cos\left(\hat{x}\right)}e^{-i\frac{\pi }{2}{\hat{n}}_{x}^{2}}e^{iK_{1}\sin\left(\hat{x}\right)},$$

(10) $${\hat{U}}_{y}=e^{i\frac{\pi }{2}{\hat{n}}_{y}^{2}}e^{-iK_{4}\cos\left(\hat{y}\right)}e^{-i\frac{\pi }{2}{\hat{n}}_{y}^{2}}e^{iK_{3}\sin\left(\hat{y}\right)}.$$

By solving the Floquet eigenvalue equations ${\hat{U}}_{x}|\psi_{x}\rangle =e^{-iE_{x}}|\psi_{x}\rangle$ and ${\hat{U}}_{y}|\psi_{y}\rangle =e^{-iE_{y}}|\psi_{y}\rangle$, we could obtain the eigenstates $\left|\psi \rangle =\right|\psi_{x}\rangle ⊗|\psi_{y}\rangle$ of $\hat{U}$ with eigenphases (quasienergies) $E=(E_{x}+E_{y})$ mod 2π. This observation immediately allows us to deduce the possible origin of higher-order topology in the 2D on-resonance double-kicked lattice. That is, if $|\psi_{x}\rangle$ and $|\psi_{y}\rangle$ are edge modes of ${\hat{U}}_{x}$ and ${\hat{U}}_{y}$ with eigenphases $\left(E_{x},E_{y}\right)=\left(0,0\right)$ or $\left(E_{x},E_{y}\right)=\left(\pi ,\pi \right)$, they will be coupled to form a corner mode of $\hat{U}$ with eigenphase $E=E_{x}+E_{y}=0$, i.e., a Floquet corner zero mode. Similarly, if $|\psi_{x}\rangle$ and $|\psi_{y}\rangle$ are edge modes of ${\hat{U}}_{x}$ and ${\hat{U}}_{y}$ with eigenphases $\left(E_{x},E_{y}\right)=\left(0,\pi \right)$ or $\left(E_{x},E_{y}\right)=\left(\pi ,0\right)$, they will be coupled to form a corner mode of $\hat{U}$ with eigenphase $E=E_{x}+E_{y}=\pi$, i.e., a Floquet corner π mode. These are the two types of topological corner modes that could appear in Floquet HOTPs of our model, and their numbers are determined by the numbers of edge modes in the subsystems described by ${\hat{U}}_{x}$ and ${\hat{U}}_{y}$, which are further determined by the winding numbers of ${\hat{U}}_{x}$ and ${\hat{U}}_{y}$ according to the principle of bulk-edge correspondence [124,125]. In the following section, we construct the bulk topological invariants for Floquet HOTPs in the 2D on-resonance double kicked lattice based on these analysis and establish the bulk topological phase diagram of the system.

## 3. Topological Invariants and Phase Diagram

Since the Floquet operator $\hat{U}$ in Equation (8) possesses a tensor product structure, its spectrum and eigenstates are known once the eigenvalue equations ${\hat{U}}_{j}|\psi_{j}\rangle =e^{-iE_{j}}|\psi_{j}\rangle$ for j=x,y are solved. Inserting the identity operators $I_{j}=\sum_{j}|n_{j}\rangle \langle n_{j}|$ in the momentum space, and performing Fourier transforms from the momentum to quasiposition (the conserved quantity due to the translational symmetry $n_{j}\to n_{j}+2$ in the momentum lattice) representation, the Floquet operator ${\hat{U}}_{j}$ can be expressed in the form of

(11) $${\hat{U}}_{j}=\sum_{\theta_{j}}|\theta_{j}\rangle U_{j}\left(\theta_{j}\right)\langle \theta_{j}|,$$

where $\left\{|\theta_{j}\rangle \right\}$ is the eigenbasis of quasiposition with $\theta_{j}\in \left[0,2\pi \right)$ and j=x,y. Explicitly, the Floquet matrices $U_{x}\left(\theta_{x}\right)$ and $U_{y}\left(\theta_{y}\right)$ are given by

(12) $$U_{x}\left(\theta_{x}\right)=e^{i\frac{\pi }{4}\sigma_{z}}e^{-iK_{2}\left(\cos\frac{\theta_{x}}{2}\sigma_{x}+\sin\frac{\theta_{x}}{2}\sigma_{y}\right)}e^{-i\frac{\pi }{4}\sigma_{z}}e^{iK_{1}\left(\cos\frac{\theta_{x}}{2}\sigma_{x}+\sin\frac{\theta_{x}}{2}\sigma_{y}\right)},$$

(13) $$U_{y}\left(\theta_{y}\right)=e^{i\frac{\pi }{4}\tau_{z}}e^{-iK_{4}\left(\cos\frac{\theta_{y}}{2}\tau_{x}+\sin\frac{\theta_{y}}{2}\tau_{y}\right)}e^{-i\frac{\pi }{4}\tau_{z}}e^{iK_{3}\left(\cos\frac{\theta_{y}}{2}\tau_{x}+\sin\frac{\theta_{y}}{2}\tau_{y}\right)},$$

with shorthand notations

(14) $$K_{1}\equiv K_{1}\sin\frac{\theta_{x}}{2},\;K_{2}\equiv K_{2}\cos\frac{\theta_{x}}{2},$$

(15) $$K_{3}\equiv K_{3}\sin\frac{\theta_{y}}{2},\;K_{4}\equiv K_{4}\cos\frac{\theta_{y}}{2}.$$

Here, $\sigma_{x,y,z}$ and $\tau_{x,y,z}$ are Pauli matrices acting on two sublattice degrees of freedom along the *x* and *y* directions in the momentum lattice (see [121,122] for derivation details of $U_{j}\left(\theta_{j}\right)$ for 1D descendant models of the 2D on-resonance double-kicked lattice). The standard characterization of 1D Floquet topological phases is achieved by introducing a pair of symmetric time frames upon similarity transformations [124,125]. For our model, there are two symmetric time frames for both $U_{x}\left(\theta_{x}\right)$ and $U_{y}\left(\theta_{y}\right)$. Putting together, there are in total four such time frames for the Floquet matrix $U\left(\theta_{x},\theta_{y}\right)=U_{x}\left(\theta_{x}\right)⊗U_{y}\left(\theta_{y}\right)$ of the 2D system. In these time frames, $U(\theta_{x},\theta_{y})$ takes the form

(16) $$U_{\alpha \beta }\left(\theta_{x},\theta_{y}\right)=U_{\alpha }\left(\theta_{x}\right)⊗U_{\beta }\left(\theta_{y}\right),$$

where α=1,2, β=3,4, and

(17) $$U_{1}\left(\theta_{x}\right)=FG,\;U_{2}\left(\theta_{x}\right)=GF,$$

(18) $$U_{3}\left(\theta_{y}\right)=F^{\prime }G^{\prime },\;U_{4}\left(\theta_{y}\right)=G^{\prime }F^{\prime }.$$

The auxiliary matrices *F*, *G*, $F^{\prime }$ and $G^{\prime }$ are explicitly given by (see [121,122] for derivation details of these matrices for 1D descendant models of our system)

(19) $$F\equiv e^{i\frac{K_{1}}{2}\left(\cos\frac{\theta_{x}}{2}\sigma_{x}+\sin\frac{\theta_{x}}{2}\sigma_{y}\right)}e^{i\frac{\pi }{4}\sigma_{z}}e^{-i\frac{K_{2}}{2}\left(\cos\frac{\theta_{x}}{2}\sigma_{x}+\sin\frac{\theta_{x}}{2}\sigma_{y}\right)},$$

(20) $$G\equiv e^{-i\frac{K_{2}}{2}\left(\cos\frac{\theta_{x}}{2}\sigma_{x}+\sin\frac{\theta_{x}}{2}\sigma_{y}\right)}e^{-i\frac{\pi }{4}\sigma_{z}}e^{i\frac{K_{1}}{2}\left(\cos\frac{\theta_{x}}{2}\sigma_{x}+\sin\frac{\theta_{x}}{2}\sigma_{y}\right)},$$

(21) $$F^{\prime }\equiv e^{i\frac{K_{3}}{2}\left(\cos\frac{\theta_{y}}{2}\tau_{x}+\sin\frac{\theta_{y}}{2}\tau_{y}\right)}e^{i\frac{\pi }{4}\tau_{z}}e^{-i\frac{K_{4}}{2}\left(\cos\frac{\theta_{y}}{2}\tau_{x}+\sin\frac{\theta_{y}}{2}\tau_{y}\right)},$$

(22) $$G^{\prime }\equiv e^{-i\frac{K_{4}}{2}\left(\cos\frac{\theta_{y}}{2}\tau_{x}+\sin\frac{\theta_{y}}{2}\tau_{y}\right)}e^{-i\frac{\pi }{4}\tau_{z}}e^{i\frac{K_{3}}{2}\left(\cos\frac{\theta_{y}}{2}\tau_{x}+\sin\frac{\theta_{y}}{2}\tau_{y}\right)}.$$

With these considerations, it is straightforward to verify that $U_{\alpha \beta }\left(\theta_{x},\theta_{y}\right)$ possesses the chiral symmetry $\Gamma =\sigma_{z}⊗\tau_{z}$ for all α=1,2 and β=3,4, in the sense that $\Gamma^{2}=1$ and

(23) $$\Gamma U_{\alpha \beta }\left(\theta_{x},\theta_{y}\right)\Gamma =U_{\alpha \beta }^{†}\left(\theta_{x},\theta_{y}\right).$$

This symmetry then allows us to characterize the Floquet HOTPs of our system by integer topological invariants [121,124,125].

To construct these topological numbers for our system, we take the Taylor expansion for each term of the Floquet matrices in Equations (17) and (18), and recombine the relevant terms. The resulting Floquet matrices take the forms

(24) $$U_{\alpha }\left(\theta_{x}\right)=e^{-iE_{x}\left(\theta_{x}\right)\left[n_{\alpha x}\left(\theta_{x}\right)\sigma_{x}+n_{\alpha y}\left(\theta_{x}\right)\sigma_{y}\right]},$$

(25) $$U_{\beta }\left(\theta_{y}\right)=e^{-iE_{y}\left(\theta_{y}\right)\left[n_{\beta x}\left(\theta_{y}\right)\tau_{x}+n_{\beta y}\left(\theta_{y}\right)\tau_{y}\right]},$$

where α=1,2 and β=3,4. The eigenphase dispersions $E_{x}\left(\theta_{x}\right)$ and $E_{y}\left(\theta_{y}\right)$ along the two different dimensions are given by

(26) $$E_{x}\left(\theta_{x}\right)=\arccos\left(\cos K_{1}\cos K_{2}\right),$$

(27) $$E_{y}\left(\theta_{y}\right)=\arccos\left(\cos K_{3}\cos K_{4}\right).$$

The explicit expressions of unit vectors $\left[n_{\alpha x}\left(\theta_{x}\right),n_{\alpha y}\left(\theta_{x}\right)\right]$ and $\left[n_{\beta x}\left(\theta_{y}\right),n_{\beta y}\left(\theta_{y}\right)\right]$ are summarized in Appendix B. In the quasiposition representation, the 2D on-resonance double-kicked lattice then possesses four bulk eigenphase (quasienergy) bands, whose dispersion relations are given by

(28) $$E_{ss^{\prime }}\left(\theta_{x},\theta_{y}\right)=sE_{x}\left(\theta_{x}\right)+s^{\prime }E_{y}\left(\theta_{y}\right),$$

where $s,s^{\prime }=\pm$. The system could undergo topological phase transitions when these bands touch and separate at the quasienergies zero and π.

In previous studies [121,122], it has been demonstrated that a 1D system described by the Floquet operator $U_{\alpha }\left(\theta_{x}\right)$ or $U_{\beta }\left(\theta_{y}\right)$ possesses a topological winding number

(29) $$w_{\nu }=\int_{0}^{2\pi }\frac{d\theta_{j}}{2\pi }\partial_{\theta_{j}}\varphi_{\nu }\left(\theta_{j}\right),$$

where ν={α,β}, j=x,y and the winding angle $\varphi_{\nu }\left(\theta_{j}\right)$ in the ν’s time frame is defined as

(30) $$\varphi_{\nu }\left(\theta_{j}\right)\equiv \arctan\left[n_{\nu y}\left(\theta_{j}\right)/n_{\nu x}\left(\theta_{j}\right)\right].$$

Using these winding numbers, we can further construct two pairs of invariants $\left(w_{0x},w_{\pi x}\right)$ and $\left(w_{0y},w_{\pi y}\right)$ for the Floquet subsystems described by ${\hat{U}}_{x}$ and ${\hat{U}}_{y}$, respectively [124,125]. They are related to the values of $w_{\nu }$ through the relations

(31) $$w_{0x}=\frac{w_{1}+w_{2}}{2},\;w_{\pi x}=\frac{w_{1}-w_{2}}{2},$$

(32) $$w_{0y}=\frac{w_{3}+w_{4}}{2},\;w_{\pi y}=\frac{w_{3}-w_{4}}{2}.$$

These invariants always take integer quantized values. They provide a complete characterization of the topological phases in 1D Floquet systems with chiral (sublattice) symmetry [124,125]. Moreover, under the open boundary condition, the invariants $w_{0x}$ ($w_{0y}$) and $w_{\pi x}$ ($w_{\pi y}$) could predict the numbers of topological edge modes with quasienergies zero and π in the subsystem described by ${\hat{U}}_{x}$ (${\hat{U}}_{y}$) [121,122], and therefore also capturing the bulk-edge correspondence of these Floquet subsystems.

For our 2D system, the Floquet HOTPs can be characterized by appropriate combinations of these 1D topological numbers. Specially, referring to our analysis on how the zero and π Floquet edge modes can be coupled to form corner modes in the last section, we introduce a pair of topological invariants $\left(w_{0},w_{\pi }\right)$ for the 2D on-resonance double-kicked lattice, which are defined as

(33) $$w_{0}\equiv |w_{0x}w_{0y}\left|+\right|w_{\pi x}w_{\pi y}|,$$

(34) $$w_{\pi }\equiv |w_{0x}w_{\pi y}\left|+\right|w_{\pi x}w_{0y}|.$$

It is clear that $(w_{0},w_{\pi })\in Z\times Z$ due to the quantized nature of $w_{0j}$ and $w_{\pi j}$ (j=x,y). Furthermore, as demonstrated in Section 5, the values of $w_{0}$ and $w_{\pi }$ could correctly count the numbers of Floquet corner modes with quasienergies zero and π in the momentum space of our system. Therefore, the invariants $\left(w_{0},w_{\pi }\right)$ could provide us with a complete characterization of Floquet HOTPs in the 2D on-resonance double-kicked lattice and other chiral symmetric 2D lattice models whose Floquet operators can be expressed in the form of $\hat{U}={\hat{U}}_{x}⊗{\hat{U}}_{y}$. We also notice that $\left(w_{0},w_{\pi }\right)\neq \left(0,0\right)$ only when the subsystems described by ${\hat{U}}_{x}$ and ${\hat{U}}_{y}$ are both topologically nontrivial. The Floquet HOTPs of the 2D on-resonance double-kicked lattice are thus originated from the nontrivial cooperation of topological natures of the two subsystems in lower dimensions.

**Figure 1:**
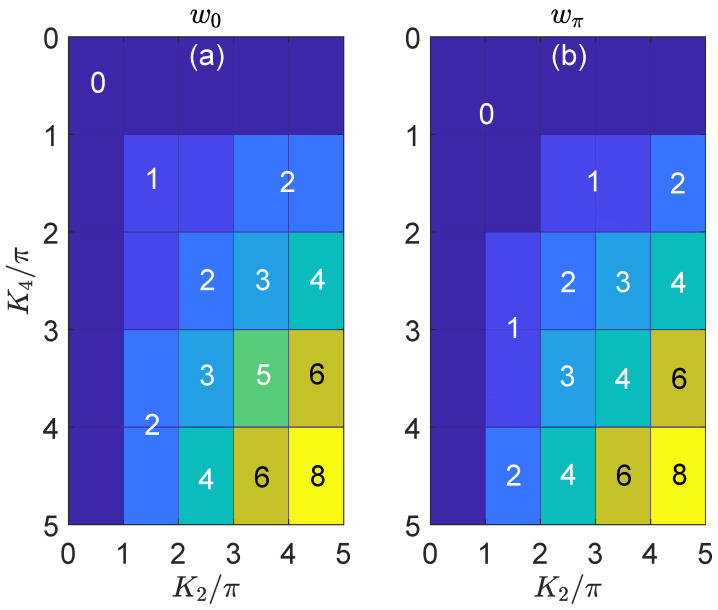
Topological phase diagram of the 2D on-resonance double-kicked lattice versus the kicking strengths $K_{2}$ and $K_{4}$. The other system parameters are set as $K_{1}=K_{3}=0.5\pi$. Different Floquet HOTPs are distinguished by different colors in each panel. The values of $w_{0}$ ($w_{\pi }$) for different topological phases are obtained from Equation (33) [(34)] and denoted explicitly in (**a**) [(**b**)].

In the remaining part of this section, based on the evaluation of invariants $w_{0}$ and $w_{\pi }$ in Equations (33) and (34), we present topological phase diagrams of the 2D on-resonance double-kicked lattice for two typical situations. In the first case, we show the phase diagram of the system with respect to the kicking strengths $\left(K_{2},K_{4}\right)$ in Figure 1. We observe that with the increase of these kicking strengths, a series of topological phase transitions can be induced, which each of them being accompanied by the quantized jump of $w_{0}$ or $w_{\pi }$. At large values of $\left(K_{2},K_{4}\right)$, we further obtain rich Floquet HOTPs characterized by large values of $\left(w_{0},w_{\pi }\right)$. It is not hard to verify that there is no upper bound for the values of these invariants if the kicking strengths keep increasing. Therefore, the 2D on-resonance double-kicked lattice serves as a good candidate to generate Floquet second-order topological phases in momentum space with arbitrarily large topological invariants. Besides, according to the tensor product structure of Floquet operator in Equation (16), the boundaries separating different Floquet HOTPs are determined by the gapless conditions along one dimension of the lattice. More explicitly, these phase boundaries are determined by the conditions $\cos[E_{x}\left(\theta_{x}\right)]=\pm 1$ and $\cos[E_{y}\left(\theta_{y}\right)]=\pm 1$, or equivalently

(35) $$\frac{m_{1}^{2}}{K_{1}^{2}}+\frac{m_{2}^{2}}{K_{2}^{2}}=\frac{1}{\pi^{2}},\;\frac{m_{3}^{2}}{K_{3}^{2}}+\frac{m_{4}^{2}}{K_{4}^{2}}=\frac{1}{\pi^{2}},$$

with $m_{i}\in Z$ and $|m_{i}\pi /K_{i}|\leq 1$ for i=1,2,3,4. In Figure 1, our choice of system parameters yield $m_{1}=m_{3}=0$, and the phase boundaries are reduced to straight lines according to Equation (35), which is also consistent with the numerical results.

**Figure 2:**
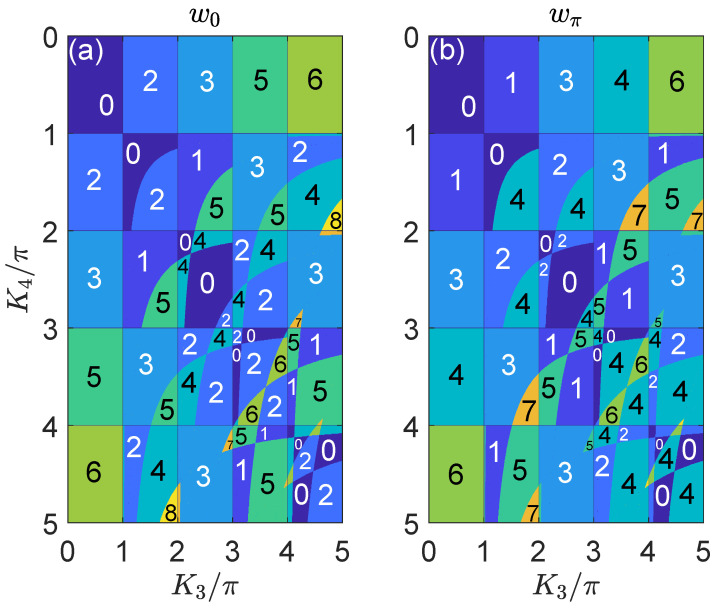
Topological phase diagram of the 2D on-resonance double-kicked lattice with respect to the kicking strengths $K_{3}$ and $K_{4}$. The other system parameters are set as $\left(K_{1},K_{2}\right)=\left(0.5\pi ,3.5\pi \right)$. Different Floquet HOTPs are distinguished by different colors in both figure panels. The values of $w_{0}$ and $w_{\pi }$ for each topological phase are obtained from Equations (33) and (34), which are presented explicitly in figure panels (**a**) and (**b**), respectively.

In the second case, we present the phase diagram of the 2D on-resonance double-kicked lattice versus kicking strengths $\left(K_{3},K_{4}\right)$ in Figure 2. With the increase of these kicking strengths, we also observe rich Floquet HOTPs featured by large invariants $\left(w_{0},w_{\pi }\right)$, together with multiple transitions between these phases followed by quantized changes of $\left(w_{0},w_{\pi }\right)$. Numerically, we have also checked the phase diagrams of the system versus any one pair of kicking parameters $\left(K_{i},K_{i^{\prime }}\right)$, with the other pair being fixed for $i,i^{\prime }=1,2,3,4$, and obtain similar patterns for the topological phases and phase transitions. Therefore, we conclude that the 2D on-resonance double-kicked lattice indeed possesses rich Floquet HOTPs, which are characterized by a pair integer topological invariants $\left(w_{0},w_{\pi }\right)$. These invariants could not only predict the numbers of Floquet zero and π corner modes in the system under open boundary conditions, but also be detectable experimentally from the dynamics of wavepackets, as presented in the following sections.

## 4. Mean Chiral Displacements

In this section, we sketch a dynamical approach that can be used to probe the invariants $\left(w_{0},w_{\pi }\right)$ of Floquet HOTPs in our system. This approach is based on the detection of a 2D extension of the time-averaged mean chiral displacement, which is introduced in [82,95]. In a 2D lattice (either in position or in momentum space), we define the chiral displacement operator of the dynamical evolution in the time frame (α,β) as

(36) $${\hat{C}}_{\alpha \beta }\left(t\right)={\hat{U}}_{\alpha \beta }^{-t}\left({\hat{N}}_{x}⊗\Gamma_{x}\right)⊗\left({\hat{N}}_{y}⊗\Gamma_{y}\right){\hat{U}}_{\alpha \beta }^{t}.$$

Here, *t* counts the number of evolution periods. The Floquet operator ${\hat{U}}_{\alpha \beta }={\hat{U}}_{\alpha }⊗{\hat{U}}_{\beta }$, as defined in Equation (16) for α=1,2 and β=3,4. ${\hat{N}}_{x}$ and ${\hat{N}}_{y}$ are unit-cell position operators along *x* and *y* directions of the lattice. $\Gamma_{x}$ and $\Gamma_{y}$ describe chiral symmetries of the descendant systems ${\hat{U}}_{\alpha }$ and ${\hat{U}}_{\beta }$ along *x* and *y* directions, respectively. For our 2D on-resonance double-kicked lattice, we have $\Gamma_{x}=\sigma_{z}$ and $\Gamma_{y}=\tau_{z}$, whose tensor product gives the chiral symmetry operator Γ of the system.

To extract the topological winding numbers of ${\hat{U}}_{\alpha \beta }$, we initialize the system in a fully polarized state at the central unit cell of the lattice. The initial state vector then takes the form

(37) $$|\psi_{0}\rangle =|0_{x}{\rangle ⊗|↑}_{x}\rangle ⊗|0_{y}{\rangle ⊗|↑}_{y}\rangle ,$$

where $|0_{x}\rangle$ ($|0_{y}\rangle$) is the eigenstate of ${\hat{N}}_{x}$ (${\hat{N}}_{y}$) with eigenvalue $N_{x}=0$ ($N_{y}=0$). ${|↑}_{x}\rangle$ and ${|↑}_{y}\rangle$ are the eigenvectors of $\Gamma_{x}$ and $\Gamma_{y}$ with eigenvalues +1. After the evolution over a number of *t*’s driving periods in the time frame (α,β), the mean chiral displacement of initial state $|\psi_{0}\rangle$ is given by the expectation value

(38) $$C_{\alpha \beta }\left(t\right)=\langle \psi_{0}|{\hat{C}}_{\alpha \beta }\left(t\right)|\psi_{0}\rangle .$$

Since $|\psi_{0}\rangle$ is not an eigenstate of ${\hat{U}}_{\alpha \beta }$, $C_{\alpha \beta }\left(t\right)$ is expected to be an oscillating function of time. To extract the topological information from $C_{\alpha \beta }\left(t\right)$, we take the average of $C_{\alpha \beta }\left(t\right)$ over many driving periods, which in the long-time limit yields the stroboscopic time-averaged mean chiral displacement

(39) $${\bar{C}}_{\alpha \beta }=\lim_{t\to \infty }\frac{1}{t}\sum_{t^{\prime }=1}^{t}C_{\alpha \beta }\left(t^{\prime }\right).$$

Following the derivation steps as detailed in [82], it can be shown that for α=1,2 and β=3,4,

(40) $${\bar{C}}_{\alpha \beta }=\frac{w_{\alpha }w_{\beta }}{4}.$$

Here, $w_{\alpha }$ and $w_{\beta }$ are the winding numbers defined in Equation (29). With the help of Equations (31) and (32), we can recombine the time-averaged mean chiral displacements to obtain the products of winding numbers $w_{0x}w_{0y}$, $w_{0x}w_{\pi y}$, $w_{\pi x}w_{0y}$ and $w_{\pi x}w_{\pi y}$, which finally yield the invariants $\left(w_{0},w_{\pi }\right)$ of the Floquet HOTPs. From now on, we denote the recombined time-averaged mean chiral displacements that are related to $w_{0}$ and $w_{\pi }$ as ${\bar{C}}_{0}$ and ${\bar{C}}_{\pi }$, respectively (see Appendix C for their explicit expressions).

**Figure 3:**
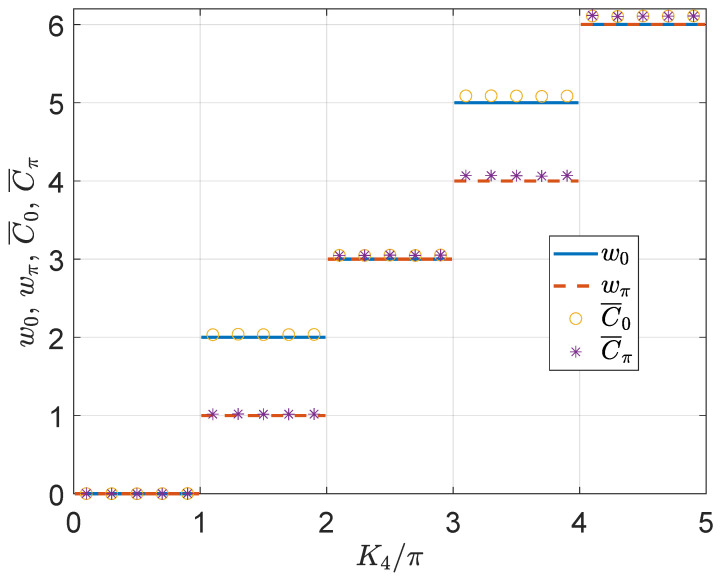
Topological invariants $\left(w_{0},w_{\pi }\right)$ and time-averaged mean chiral displacements $\left({\bar{C}}_{0},{\bar{C}}_{\pi }\right)$ of the 2D on-resonance double-kicked lattice versus the kicking strength $K_{4}$. The other system parameters are set as $K_{1}=0.5\pi$, $K_{2}=3.5\pi$ and $K_{3}=0.5\pi$. The mean chiral displacements are averaged over t=50 driving periods to generate the results for $\left({\bar{C}}_{0},{\bar{C}}_{\pi }\right)$.

To verify the relations between the time-averaged mean chiral displacements and the topological invariants of the 2D on-resonance double-kicked lattice, we compute and compare $\left(w_{0},w_{\pi }\right)$ and $\left({\bar{C}}_{0},{\bar{C}}_{\pi }\right)$ for a typical case in the remaining part of this section. In Figure 3, we present the topological invariants and mean chiral displacements with respect to the kicking strength $K_{4}$ for evolutions over t=50 driving periods. We observe that the time-averaged mean chiral displacements $\left({\bar{C}}_{0},{\bar{C}}_{\pi }\right)$ indeed take nearly quantized values in each Floquet HOTPs of the 2D on-resonance double-kicked lattice, which demonstrate the relation between them and the topological invariants of the system, i.e., $\left({\bar{C}}_{0},{\bar{C}}_{\pi }\right)=\left(w_{0},w_{\pi }\right)$ (see Appendix C for derivation details of this relation). Besides, the values of $\left({\bar{C}}_{0},{\bar{C}}_{\pi }\right)$ also possess quantized jumps around all topological phase transition points ($K_{4}=\pi ,2\pi ,3\pi ,4\pi$ in Figure 3). Therefore, the mean chiral displacements could also serve as a dynamical prob to the phase transitions between different Floquet HOTPs. The deviations of $\left({\bar{C}}_{0},{\bar{C}}_{\pi }\right)$ from quantization are due to finite-time effects, which will gradually go to zero with the increase of the number of driving periods *t*. Numerically, we checked that the time-averaged mean chiral displacements $\left({\bar{C}}_{0},{\bar{C}}_{\pi }\right)$ remain close to quantization for t=20 driving periods, which should be within reach in current or near-term experiments in photonic [126,127] and cold atom [128,129] systems.

In the following section, we demonstrate another topological signature of Floquet HOTPs, i.e., the symmetry protected bound states localized around the corners of the momentum-space lattice, and relate their numbers to the topological invariants $\left(w_{0},w_{\pi }\right)$ of the system.

## 5. Floquet Topological Corner Bound States in Continuum

A defining feature of HOTPs in *D* spatial dimensions is symmetry-protected states localized along its (D−d)-dimensional boundaries, where d>1. For a 2D lattice studied in this work, such bound states could appear at the geometric corners of the system. Moreover, since the Floquet bands of a chiral symmetric system come in positive and negative pairs ±E, they could touch and separate at the quasienergies zero and ±π. Therefore, in principle, there could be two distinct types of Floquet corner modes appearing at these quasienergies, which are called Floquet zero and π corner modes. In the 2D on-resonance double-kicked lattice described by $\hat{U}={\hat{U}}_{x}⊗{\hat{U}}_{y}$, since these corner modes are formed by the coupling between doubly degenerate edge modes of 1D descendant systems ${\hat{U}}_{x}$ and ${\hat{U}}_{y}$, their numbers $\left(N_{0},N_{\pi }\right)$ are always integer multiples of four. Furthermore, the numbers $\left(N_{0},N_{\pi }\right)$ of Floquet zero and π corner modes tend to be connected with the topological invariants $\left(w_{0},w_{\pi }\right)$ of the bulk through the relation

(41) $$\left(N_{0},N_{\pi }\right)=4\left(w_{0},w_{\pi }\right).$$

Equation (41) thus establishes the bulk-corner correspondence of Floquet HOTPs in our model and other chiral symmetric systems, whose Floquet operators can be expressed in the tensor product form of $\hat{U}={\hat{U}}_{x}⊗{\hat{U}}_{y}$. The implication of Equation (41) is demonstrated in the following with explicit numerical examples.

To investigate the spectrum and states of the system under open boundary conditions, we express the operators in Equations (9) and (10) in momentum lattice representations as (see Appendix A for details)

(42) $$\begin{array}{cc} {\hat{U}}_{x} & =e^{i\frac{\pi }{2}\sum_{n_{x}}n_{x}^{2}|n_{x}\rangle \langle n_{x}|}e^{-i\frac{K_{2}}{2}\sum_{n_{x}}(|n_{x}\rangle \langle n_{x}+1|+h.c.)}\\ & \times e^{-i\frac{\pi }{2}\sum_{n_{x}}n_{x}^{2}|n_{x}\rangle \langle n_{x}|}e^{i\frac{K_{1}}{2i}\sum_{n_{x}}(|n_{x}\rangle \langle n_{x}+1|-h.c.)}, \end{array}$$

(43) $$\begin{array}{cc} {\hat{U}}_{y} & =e^{i\frac{\pi }{2}\sum_{n_{y}}n_{y}^{2}|n_{y}\rangle \langle n_{y}|}e^{-i\frac{K_{4}}{2}\sum_{n_{y}}(|n_{y}\rangle \langle n_{y}+1|+h.c.)}\\ & \times e^{-i\frac{\pi }{2}\sum_{n_{y}}n_{y}^{2}|n_{y}\rangle \langle n_{y}|}e^{i\frac{K_{3}}{2i}\sum_{n_{y}}(|n_{y}\rangle \langle n_{y}+1|-h.c.)}. \end{array}$$

The quasienergies *E* and Floquet eigenstates |ψ〉 of the system are then obtained by solving the eigenvalue equation $\hat{U}|\psi \rangle =e^{-iE}|\psi \rangle$, where the diagonalization of $\hat{U}$ can be performed separately for ${\hat{U}}_{x}$ and ${\hat{U}}_{y}$ due to the tensor product structure of the Floquet operator $\hat{U}={\hat{U}}_{x}⊗{\hat{U}}_{y}$.

**Figure 4:**
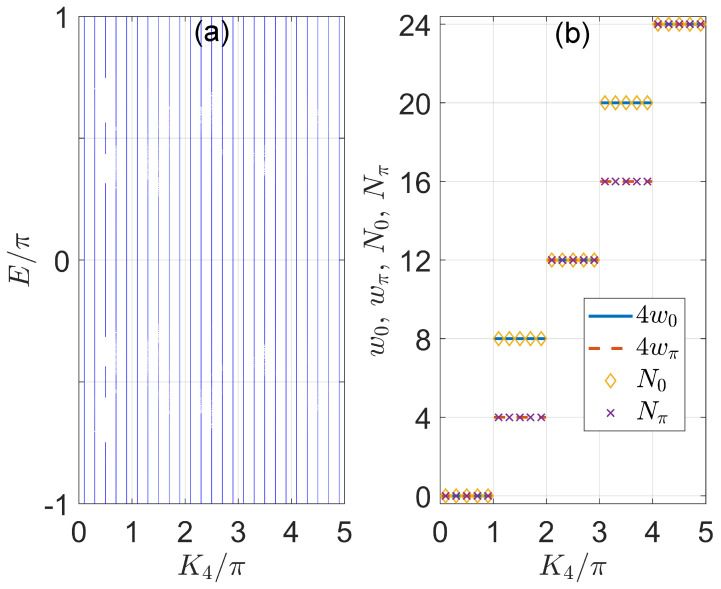
Floquet spectrum *E* in panel (**a**) and number of corner modes $\left(N_{0},N_{\pi }\right)$ in panel **b** of the 2D on-resonance double−kicked lattice versus the kicking strength $K_{4}$. The other system parameters are set as $\left(K_{1},K_{2},K_{3}\right)=\left(0.5\pi ,3.5\pi ,0.5\pi \right)$. The size of the lattice is $L_{x}=L_{y}=300$. In panel (**b**), $N_{0}$ and $N_{\pi }$ are plotted together with the bulk topological invariants $w_{0}$ and $w_{\pi }$ as obtained from Equations (33) and (34).

In Figure 4, we present the quasienergy spectrum *E* and number of Floquet corner modes $N_{0}$ and $N_{\pi }$ at zero and π quasienergies in the momentum space of our model. In Figure 4a, we observe an almost continuous Floquet spectrum with no gaps around the quasienergies E=0 and E=π. This is different from the situations usually observed in 2D static and Floquet HOTPs, where corner modes are separated from bulk states by spectral gaps. However, by evaluating the inverse participation ratio

(44) $$\mathrm{IPR}\equiv \sum_{n_{x},n_{y}}{\left|\psi \left(n_{x},n_{y}\right)\right|}^{4}$$

for all the Floquet eigenstates of $\hat{U}$, we find different numbers of corner modes $N_{0}$ and $N_{\pi }$ at the quasienergies zero and π in distinct Floquet HOTPs of the system. Their numbers are presented together with the topological invariants $w_{0}$ and $w_{\pi }$ in Figure 4b. Note that the inverse participation ratio of corner modes differ from bulk and 1D edge states of the system in their order of magnitudes, and can thus be numerically distinguished from one another. Furthermore, we observe that the relation $\left(N_{0},N_{\pi }\right)=4\left(w_{0},w_{\pi }\right)$ holds throughout the considered parameter regime, validating the bulk-corner correspondence of our model as established in Equation (41). Besides, with the increase of $K_{4}$, the system undergoes a series of topological phase transitions, yielding Floquet HOTPs with more and more zero and π corner modes. The 2D on-resonance double-kicked lattice thus provides us with a nice platform to investigate Floquet HOTPs with multiple corner states and strong topological signatures in momentum space. For completeness, we also checked the quasienergy spectrum and corner modes in other parameter regions of the system and obtained results that are consistent with the above descriptions.

**Figure 5:**
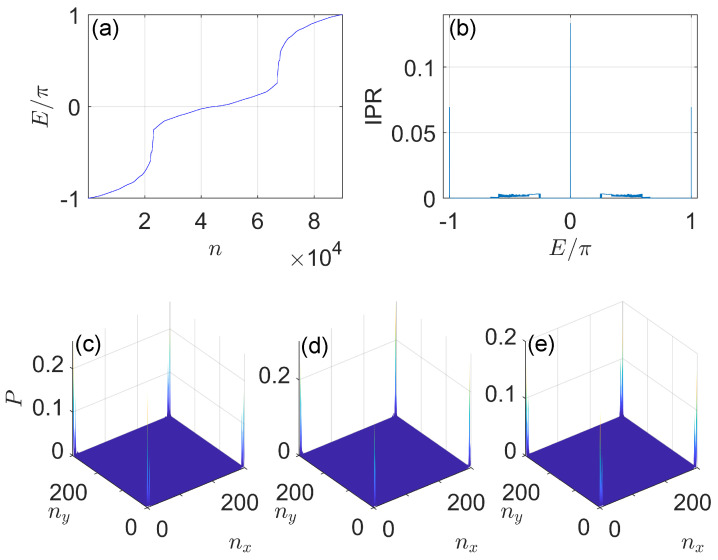
Quasienergy spectrum, inverse participation ratio and Floquet zero/π corner modes of the 2D on−resonance double−kicked lattice. System parameters are set as $K_{1}=0.5\pi$, $K_{2}=3.5\pi$, $K_{3}=0.5\pi$ and $K_{4}=1.5\pi$. The size of the lattice is $L_{x}=L_{y}=300$. (**a**) *n* denotes the index of the state. The peaks around E=0 (E=±π) in (**b**) correspond to the inverse participation ratio of zero (π) Floquet corner modes, whose probability distributions are shown in panels (**c**–**e**).

In Figure 5, we present the quasienergy spectrum, inverse participation ratio and corner modes of the system for a typical situation. In Figure 5a, we observe that the Floquet spectrum *E* spread throughout the first quasienergy Brillouin zone, and no gaps can be observed around E=0,±π. However, in Figure 5b, we find three clear peaks in the inverse participation ratio versus the quasienergy *E* around E=0 and E=±π, which indicates the existence of localized bound states in the system at these quasienergies. By investigating the data, we obtain eight (four) such bound states at E=0 (E=±π), with their probability distributions shown explicitly in Figure 5d,e [Figure 5c]. We see that all of these bound states are indeed localized around the corners of the system, and their numbers are determined precisely by the bulk-corner correspondence relation in Equation (41) for the given set of system parameters. Therefore, these corner states originate from the higher-order topology of our system. They represent topologically degenerate corner modes in momentum space of the system, which are protected by the chiral symmetry Γ. Besides, these corner modes coexist with extended bulk states at the same quasienergies. We therefore refer to them as corner-localized Floquet topological bound states in continuum, in the sense that they do not hybridize with the surrounding bulk states even without a bulk band gap. This observation also extends the scope of bulk-corner correspondence of Floquet HOTPs to more general situations, in which the symmetry-protected corner modes can not only be found in spectral gaps, but also appear within topological Floquet bands. Note in passing that the corner-localized bound states in continuum with zero energy have been discovered before in static HOTPs [130,131,132]. By contrast, the corner-localized Floquet bound states in continuum subject to different topological characterizations, and the corner bound states in continuum with quasienergy E=π are unique to Floquet HOTPs found in this work. Experimentally, the implementation of open boundary conditions might be achieved in Bose–Einstein condensates by a setup similar to the one employed in the realization of topological quantum walk in momentum space [129]. Meanwhile, by applying the mapping introduced in [121] from momentum to position space lattices, the 2D on-resonance double-kicked lattice may also be realized in real space, and the corner modes may then be probed in setups such as photonic waveguide arrays [132].

**Figure 6:**
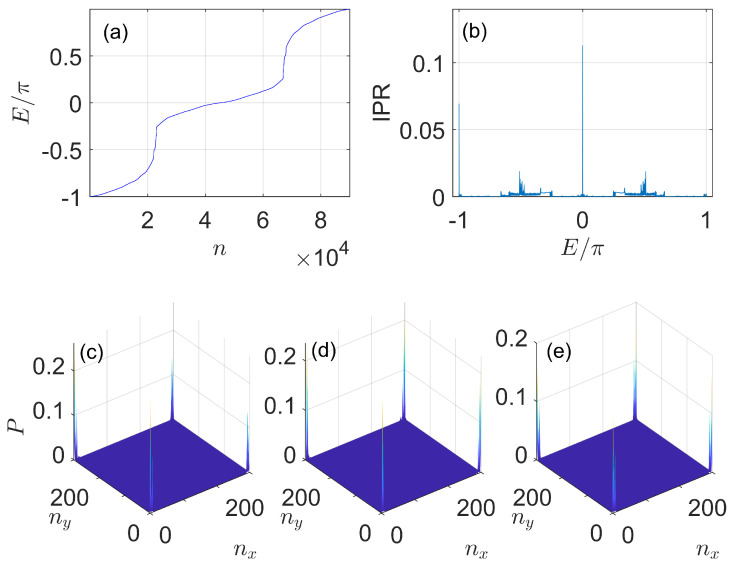
Quasienergy spectrum, inverse participation ratio and Floquet zero/π corner modes of the 2D on−resonance double−kicked lattice with hopping disorder. System parameters are set as $K_{1}=0.5\pi +r_{1}$, $K_{2}=3.5\pi +r_{2}$, $K_{3}=0.5\pi +r_{3}$ and $K_{4}=1.5\pi +r_{4}$, where $r_{1,2}$ ($r_{3,4}$) take random values in the range [−W/2,W/2] with W=0.1 at different nearest neighbor bonds along $n_{x}$ ($n_{y}$) direction. The size of the lattice is $L_{x}=L_{y}=300$. (**a**) *n* denotes the index of the state. The peaks around E=0 (E=−π) in (**b**) correspond to the inverse participation ratio of zero (π) Floquet corner modes in the presence of disorder, whose probability distributions are shown in (**c**–**e**).

To demonstrate the robustness of Floquet second-order topological phases found in our system, we add disorder to the nearest neighbor hopping amplitudes in the momentum lattice, i.e., by letting the kicking strengths $K_{1,2}\to K_{1,2}+r_{1,2}\left(n_{x}\right)$ and $K_{3,4}\to K_{3,4}+r_{3,4}\left(n_{y}\right)$ to be dependent on the lattice site indices $n_{x}$ and $n_{y}$. Here, $r_{1,2}\left(n_{x}\right)$ and $r_{3,4}\left(n_{y}\right)$ are uniformly distributed random numbers in the range [−W/2,W/2] at different site indices $\left(n_{x},n_{y}\right)$. In numerical calculations, we set W=0.1 as the strength of disorder and present a representative example of the Floquet spectrum and corner states with such hopping disorder in Figure 6. We observe that with disorder, the Floquet corner modes are still preserved, as shown in Figure 6c–e, and their quasienergies are also pinned at E=0 and E=±π. They are thus well-defined Floquet zero and π corner modes and their presence demonstrate the robustness of Floquet second-order topological phases of our system with disorder. Furthermore, the total number of corner modes with quasienergy zero (π) is eight (four), as shown in Figure 6c–e, which is precisely predicted by the bulk-corner correspondence relation in Equation (41). Therefore, the bulk topological invariants $w_{0}$ and $w_{\pi }$ introduced in Equations (33) and (34) could also describe the Floquet second-order topological phases of our system in the presence of weak hopping disorder.

## 6. Conclusions

In this work, we find rich Floquet second-order topological phases in a two-dimensional extension of the on-resonance double kicked rotor. Each of the Floquet phases is characterized by a pair of integer topological invariants $\left(w_{0},w_{\pi }\right)$, which can be extracted from the dynamics of the system in four distinct symmetric time frames. The values of invariants $w_{0}$ and $w_{\pi }$ take quantized jumps whenever the system undergoes a transition between different phases. Furthermore, Floquet second-order topological phases characterized by arbitrarily large $\left(w_{0},w_{\pi }\right)$ can be found in principle with the increase of kicking strengths. Experimentally, the invariants $\left(w_{0},w_{\pi }\right)$ could also be obtained by measuring the time-averaged mean chiral displacements of initially localized wavepackets in different time frames. Under open boundary conditions, corner-localized modes with quasienergies zero and π are found to be coexisting with extended bulk states at the same quasienergies, realizing second-order Floquet topological bound states in continuum. The numbers of these corner modes are further determined by the bulk topological invariants $\left(w_{0},w_{\pi }\right)$, leading to the bulk-corner correspondence of Floquet second-order topological phases. Experimentally, the proposed model may also be engineered in two-dimensional photonic or cold atom systems, where the Floquet corner modes could be imaged either in real-space [132] or in momentum space [129]. Putting together, we uncover a unique set of Floquet second-order topological phases in momentum space, which are featured by large topological invariants, rich phase diagrams and multiple Floquet topological corner bound states in continuum. In future studies, it would be interesting to extend our results to Floquet HOTPs in higher dimensions, different symmetry classes and superconducting systems. The possibility of realizing topological time crystals by superposing the zero and π Floquet corner modes and the potential applications of these corner bound states in Floquet quantum computing tasks also deserve more systematic explorations.

## Appendix A. Floquet Operator in Momentum Representation

In this appendix, we give the expressions of Floquet operators ${\hat{U}}_{x}$ and ${\hat{U}}_{y}$ in Equations (9) and (10) in the momentum-lattice representation and further derive their forms in the symmetric time frames.

In the momentum basis $\left\{|n_{x}\rangle \right\}$, we can express each term in Equation (9) of the main text as

(A1) $$U_{x\pm }\equiv e^{\pm \left(\pi /2\right){\hat{n}}_{x}^{2}}=e^{\pm i\pi /4}e^{\pm i\left(\pi /4\right)\sum_{n_{x}\in Z}(|2n_{x}-1\rangle \langle 2n_{x}-1|-|2n_{x}\rangle \langle 2n_{x}|)},$$

(A2) $$U_{x1}\equiv e^{iK_{1}\sin\left(\hat{x}\right)}=e^{i\left(K_{1}/2i\right)\sum_{n_{x}\in Z}(|2n_{x}-1\rangle \langle 2n_{x}|+|2n_{x}\rangle \langle 2n_{x}+1|-H.c.)},$$

(A3) $$U_{x2}\equiv e^{-iK_{2}\cos\left(\hat{x}\right)}=e^{-i\left(K_{2}/2\right)\sum_{n_{x}\in Z}(|2n_{x}-1\rangle \langle 2n_{x}|+|2n_{x}\rangle \langle 2n_{x}+1|+H.c.)},$$

where we insert the identity ${\hat{I}}_{x}=\sum_{n_{x}\in Z}|n_{x}\rangle \langle n_{x}|$ in order to arrive at each expression, as well as write the basis with even and odd lattice indices separately.

Since Equation (9) is invariant under the translation over two sites in ${\hat{n}}_{x}$, we could decompose the momentum space lattice into two chains containing only odd and even sites, denoted by sublattice indices *A* (for odd sites) and *B* (for even sites). A unit cell of the momentum space lattice now contains two sublattice sites. We can then introduce Pauli matrices in the sublattice representation as

(A4) $$\sigma_{x}=\left|A\rangle \langle B\right|+|B\rangle \langle A|,\;\sigma_{y}=i(|B\rangle \langle A|-|A\rangle \langle B|),\;\sigma_{z}=\left|A\rangle \langle A\right|-|B\rangle \langle B|.$$

The raising and lowering operators in sublattice basis can also be written as

(A5) $$\sigma_{\pm }=\frac{\sigma_{x}\pm i\sigma_{y}}{2}.$$

In such a bipartite lattice representation, Equations (A1)–(A3) can be expressed as

(A6) $$U_{x\pm }=e^{\pm i\pi /4}e^{\pm i\left(\pi /4\right)\sum_{\ell_{x}\in Z}|\ell_{x}\rangle \langle \ell_{x}|\sigma_{z}},$$

(A7) $$U_{x1}=e^{i\left(K_{1}/2i\right)\sum_{\ell_{x}\in Z}(|\ell_{x}\rangle \langle \ell_{x}|\sigma_{+}+|\ell_{x}\rangle \langle \ell_{x}+1|\sigma_{-}-H.c.)},$$

(A8) $$U_{x2}=e^{-i\left(K_{2}/2\right)\sum_{\ell_{x}\in Z}(|\ell_{x}\rangle \langle \ell_{x}|\sigma_{+}+|\ell_{x}\rangle \langle \ell_{x}+1|\sigma_{-}+H.c.)},$$

where $\ell_{x}$ is the unit cell index along ${\hat{n}}_{x}$-direction in momentum space, and we make the identifications

(A9) $$|2n_{x}-1\rangle \to |\ell_{x},A\rangle =|\ell_{x}\rangle |A\rangle ,\;|2n_{x}\rangle \to |\ell_{x},B\rangle =|\ell_{x}\rangle |B\rangle$$

for $n_{x}\in Z$. In momentum space, we can then write Equation (9) as

(A10) $${\hat{U}}_{x}=U_{x+}U_{x2}U_{x-}U_{x1}.$$

Following the same procedure, we can also express Equation (10) in momentum representation as

(A11) $${\hat{U}}_{y}=U_{y+}U_{y4}U_{y-}U_{y3},$$

where

(A12) $$U_{y\pm }=e^{\pm i\pi /4}e^{\pm i\left(\pi /4\right)\sum_{\ell_{y}\in Z}|\ell_{y}\rangle \langle \ell_{y}|\tau_{z}},$$

(A13) $$U_{y3}=e^{i\left(K_{3}/2i\right)\sum_{\ell_{y}\in Z}(|\ell_{y}\rangle \langle \ell_{y}|\tau_{+}+|\ell_{y}\rangle \langle \ell_{y}+1|\tau_{-}-H.c.)},$$

(A14) $$U_{y4}=e^{-i\left(K_{4}/2\right)\sum_{\ell_{y}\in Z}(|\ell_{y}\rangle \langle \ell_{y}|\tau_{+}+|\ell_{y}\rangle \langle \ell_{y}+1|\tau_{-}+H.c.)},$$

with $\tau_{\pm }=\left(\tau_{x}\pm i\tau_{y}\right)/2$ and $\tau_{x,y,z}$ being Pauli matrices acting in the subspace of the two sublattices along ${\hat{n}}_{y}$-direction. The Floquet operators in quasiposition representations, as given by Equations (12) and (13) of the main text, can further be obtained by performing the Fourier transform $|\ell_{x,y}\rangle =\left(1/\sqrt{N_{x,y}}\right)\sum_{\theta_{x,y}}e^{-i\theta_{x,y}\ell_{x,y}}|\theta_{x,y}\rangle$, with $N_{x,y}$ being the number of unit cells along ${\hat{n}}_{x}$- and ${\hat{n}}_{y}$-directions.

To obtain the Floquet operators ${\hat{U}}_{\alpha }$ and ${\hat{U}}_{\beta }$ in symmetric time frames α=1,2, β=3,4 and in the momentum-lattice representation, which are used in the calculation of the chiral displacement in Equation (36), one can simply perform similarity transformations to Equations (A10) and (A11), yielding

(A15) $${\hat{U}}_{1}=U_{x1}^{1/2}U_{x+}U_{x2}U_{x-}U_{x1}^{1/2},$$

(A16) $${\hat{U}}_{2}=U_{x2}^{1/2}U_{x-}U_{x1}U_{x+}U_{x2}^{1/2},$$

(A17) $${\hat{U}}_{3}=U_{y3}^{1/2}U_{y+}U_{y4}U_{y-}U_{y3}^{1/2},$$

(A18) $${\hat{U}}_{4}=U_{y4}^{1/2}U_{y-}U_{y3}U_{y+}U_{y4}^{1/2}.$$

## Appendix B. Components of the Effective Hamiltonian

In this appendix, we summarize the explicit expressions of unit vectors $\left[n_{\alpha x}\left(\theta_{x}\right),n_{\alpha y}\left(\theta_{x}\right)\right]$ and $\left[n_{\beta x}\left(\theta_{y}\right),n_{\beta y}\left(\theta_{y}\right)\right]$ for α=1,2 and β=3,4 in Equations (24) and (25) of the main text, respectively. Following the derivation steps as sketched in the main text and detailed in [121,122] for 1D descendant models of the 2D on-resonance double-kicked lattice, the components of these unit vectors are found to be

(A19) $$n_{1x}\left(\theta_{x}\right)=-\frac{\cos\frac{\theta_{x}}{2}\sin K_{1}\cos K_{2}-\sin\frac{\theta_{x}}{2}\sin K_{2}}{\sin[E_{x}\left(\theta_{x}\right)]},$$

(A20) $$n_{1y}\left(\theta_{x}\right)=-\frac{\sin\frac{\theta_{x}}{2}\sin K_{1}\cos K_{2}+\cos\frac{\theta_{x}}{2}\sin K_{2}}{\sin[E_{x}\left(\theta_{x}\right)]},$$

(A21) $$n_{2x}\left(\theta_{x}\right)=+\frac{\cos\frac{\theta_{x}}{2}\cos K_{1}\sin K_{2}+\sin\frac{\theta_{x}}{2}\sin K_{1}}{\sin[E_{x}\left(\theta_{x}\right)]},$$

(A22) $$n_{2y}\left(\theta_{x}\right)=+\frac{\sin\frac{\theta_{x}}{2}\cos K_{1}\sin K_{2}-\cos\frac{\theta_{x}}{2}\sin K_{1}}{\sin[E_{x}\left(\theta_{x}\right)]},$$

and

(A23) $$n_{3x}\left(\theta_{y}\right)=-\frac{\cos\frac{\theta_{y}}{2}\sin K_{3}\cos K_{4}-\sin\frac{\theta_{y}}{2}\sin K_{4}}{\sin[E_{y}\left(\theta_{y}\right)]},$$

(A24) $$n_{3y}\left(\theta_{y}\right)=-\frac{\sin\frac{\theta_{y}}{2}\sin K_{3}\cos K_{4}+\cos\frac{\theta_{y}}{2}\sin K_{4}}{\sin[E_{y}\left(\theta_{y}\right)]},$$

(A25) $$n_{4x}\left(\theta_{y}\right)=+\frac{\cos\frac{\theta_{y}}{2}\cos K_{3}\sin K_{4}+\sin\frac{\theta_{y}}{2}\sin K_{3}}{\sin[E_{y}\left(\theta_{y}\right)]},$$

(A26) $$n_{4y}\left(\theta_{y}\right)=+\frac{\sin\frac{\theta_{y}}{2}\cos K_{3}\sin K_{4}-\cos\frac{\theta_{y}}{2}\sin K_{3}}{\sin[E_{y}\left(\theta_{y}\right)]}.$$

Here, $K_{1,2}$ and $K_{3,4}$ are given by Equations (14) and (15) of the main text, respectively.

## Appendix C. Relations between Mean Chiral Displacements and Topological Invariants

In this appendix, we present the explicit relationship between the time-averaged mean chiral displacements and the topological invariants of our model. Following Equations (31), (32) and (40) in the main text, it is straightforward to verify the following equalities between the time-averaged mean chiral displacements and winding numbers in different combinations of symmetric frames

(A27) $${\bar{C}}_{13}+{\bar{C}}_{14}+{\bar{C}}_{23}+{\bar{C}}_{24}=w_{0x}w_{0y},$$

(A28) $${\bar{C}}_{13}-{\bar{C}}_{14}-{\bar{C}}_{23}+{\bar{C}}_{24}=w_{\pi x}w_{\pi y},$$

(A29) $${\bar{C}}_{13}-{\bar{C}}_{14}+{\bar{C}}_{23}-{\bar{C}}_{24}=w_{0x}w_{\pi y},$$

(A30) $${\bar{C}}_{13}+{\bar{C}}_{14}-{\bar{C}}_{23}-{\bar{C}}_{24}=w_{\pi x}w_{0y}.$$

Combining these equations with the definitions of $w_{0}$ and $w_{\pi }$ in Equations (33) and (34) of the main text, we find

(A31) $${\bar{C}}_{0}\equiv |{\bar{C}}_{13}+{\bar{C}}_{14}+{\bar{C}}_{23}+{\bar{C}}_{24}\left|+\right|{\bar{C}}_{13}-{\bar{C}}_{14}-{\bar{C}}_{23}+{\bar{C}}_{24}|=w_{0},$$

(A32) $${\bar{C}}_{\pi }\equiv |{\bar{C}}_{13}-{\bar{C}}_{14}+{\bar{C}}_{23}-{\bar{C}}_{24}\left|+\right|{\bar{C}}_{13}+{\bar{C}}_{14}-{\bar{C}}_{23}-{\bar{C}}_{24}|=w_{\pi }.$$

Therefore, by measuring the time-averaged mean chiral displacements ${\bar{C}}_{\alpha \beta }$ for α=1,2 and β=3,4, we could obtain the invariants $\left(w_{0},w_{\pi }\right)$ of Floquet HOTPs from the wavepacket dynamics in different time frames.
